# Treatment of Malignant Cholera

**Published:** 1849

**Authors:** 


### Article IV.—*Treatment of Malignant Cholera.*

The following comparison of various modes of treating malignant cholera, is taken from a review of several recent writings on this disease in the Monthly Journal, 1849:—

*Blood-letting.*—In reference to this, the evidence is conflicting. It has been employed with alleged benefit in all stages of the disease in this country and in India. In the early stage it has been effectual in relieving the feeling of oppression on the chest. Its effects on the mortality is not evident. Dr. Robertson, in his statistical account of the cholera in Edinburgh, during the present epidemic, states that he has in many instances prevented collapse by this measure.

*Stimulants.*—According to M. Ross' tables, stimulants given to any extent, appear to have been injurious.

*Opium.*—There seems no reason to doubt its efficacy in the early stage, but according to the tables above mentioned, it does not diminish the mortality.

*Mercury* has not been followed by remarkable success, in this country, except in the hands of Drs. Ayres and Peacock, both of whom use it without stimulants. In their experience the mortality was reduced to thirty-one per cent. Dr. Fleming advises the use of a solution of the bichloride, as more readily absorbed.

*Tartar Emetic* in small doses, with cold water, ad libitum, has, in the Droitwich Asylum, afforded the largest per centage of cures, the deaths being only four in twenty-four cases.

*Injections into the Veins* has afforded no satisfactory results.

*Chloroform* has been used by inhalation in thirty-seven cases. The results are inferior to those witnessed in the Droitwich Asylum, but superior to the general results exhibited in Ross' tables.

